# Illustration of *Kleda* concept in “common soil hypothesis” context

**DOI:** 10.1016/j.jaim.2023.100752

**Published:** 2026-05-13

**Authors:** H.S. Mythri, R. Nithin Krishnan

**Affiliations:** a Department of Kayachikitsa, National Institute of Ayurveda, Ministry of Ayush, Panchkula, Haryana; b Department of Roga Nidana Evum Vikruti Vijnana, Shri Dhanvantari Ayurveda College and Hospital, Chandigarh

**Keywords:** *Kleda*, *Santarpanottha Vikara*, Common soil hypothesis, Ayurveda, Oxidative stress, Metabolic syndrome

## Abstract

*Kleda* is one of the important but least explored concepts of Ayurveda. The term *Kleda* literally means ‘dampness/moisture’. *Kleda* plays an essential role in maintaining the physiology and manifesting diseases when imbalanced. It is the fundamental constituent in the pathogenesis of diseases associated with *Pitta* and *Kapha Dosha*. ‘Common soil hypothesis’ explains regarding the common prevailing substrate which is invariably involved in the manifestation of several Metabolic Disorders. There are several candidates for ‘common soil’, such as insulin resistance, vascular inflammation, and endothelial dysfunction, possessing oxidative stress and chronic inflammation as centrally participating mechanisms in all stages of diseases. This article presents a complete review on understanding Common soil hypothesis on the grounds of *Kleda* and *Santarpanottha Vyadhi* (diseases arising due to over nourishment). This review of the concept of *Kleda* has been outlined to foreground the necessity of a holistic approach in promotion of health, prevention, and management of variants of Metabolic disorders which sprout from the unified soil of *Kleda*.

The foundational principles of Ayurveda possess depth, authenticity, and a profound understanding of the diverse physiological and pathological processes within the human body. The terminologies employed in Ayurvedic texts hint at an extensive reservoir of knowledge that demands exploration for a comprehensive understanding and effective application of Ayurveda. The *Trisootra* (Three Fundamental Aphorisms) of Ayurveda encapsulates insights into the three domains of pathologic conditions, crucial for understanding disease onset, development, and management [1]. Central to this understanding is *Kleda*, an important factor influencing all the 3 aphorisms of etiology, symptomatology and treatment. It is pivotal in preserving body physiology and disease manifestation. This knowledge serves as a fundamental tool for interpreting diagnoses, predicting outcomes, managing conditions, and determining treatment responses. Despite its significance, *Kleda* remains a less explored aspect of the human body.

*Kleda* represents the body's fundamental need for moisture, deriving its name from the root “*Klid*” *dhatu*, signifying moisture or dampness. It distinctly differs from mere water due to the presence of various solutes like blood cells and proteins, although sharing similar attributes otherwise. Characteristics attributed to *Kleda* include coolness, fluidity, and lubrication.

Its physiological role relates to the transformation of consumed food *(Ahara)* and its impact on the body. *Kleda* contributes to softening food particles, aiding in their breakdown, by inducing moisture, thereby adding wetness to the ingested food [2]. Additionally, it maintains an appropriate level of oiliness *(Sneha)* within the body's tissues. *Kleda*, the moisture or liquid present between *Dhatu* (Tissue) and *Ashaya*, plays a crucial role in bodily processes. It is responsible for generating subsequent tissues and also contributes to the creation of *Kala*, an entity pivotal in tissue differentiation [3]. Any hindrance in proper formation of membrane due to abnormal *Kleda*, might lead to a decline in the distinctive functionality of tissues and tissue differentiation. Similarly fluid accumulations in spaces are also possible in pathological conditions like *Prameha* (∼Diabetes). An increased concentration of glucose initially in the extracellular fluid drives the fluid from intracellular fluid to the extracellular compartment, leading to increase in glucose concentration and osmotic diuresis. This leads to polyuria and disrupts the balance between intracellular and extracellular compartments.

The processed nutrients from digestion don't just impact tissues but also have an influence on *Kleda*. Therefore, foods rich in cold, oily, fluid, heavy qualities, can prompt the creation of imbalanced *Kleda* due to similar traits. Once the body fluid reaches the bladder, it is referred to as *Mutra* (Urine) [4]. Both urine and sweat are crucial players in maintaining *Kleda*, ensuring its optimal concentration and quality for nurturing bodily tissues. When excess, *Kleda* is expelled through urine, carrying wastes, helping to maintain the necessary physiological balance required for effective tissue nourishment. This notion is further substantiated by Acharya Charaka, who emphasizes the significance of consuming food rich in the six tastes, primarily the sweet taste. This dietary preference ultimately gives rise to the *Kleda* dominant in *Sheeta* (cool) and *Madhura* (sweet) attributes, essential for nourishing the body. Acharya Charaka observed excess *Kleda* formation or its reduced clearance, can lead to tissue looseness, an etiological factor in conditions like *Prameha* and *Kushta*. Various diseases attributed to *Kleda* are depicted in Fig. 1.

**Fig. 1:**
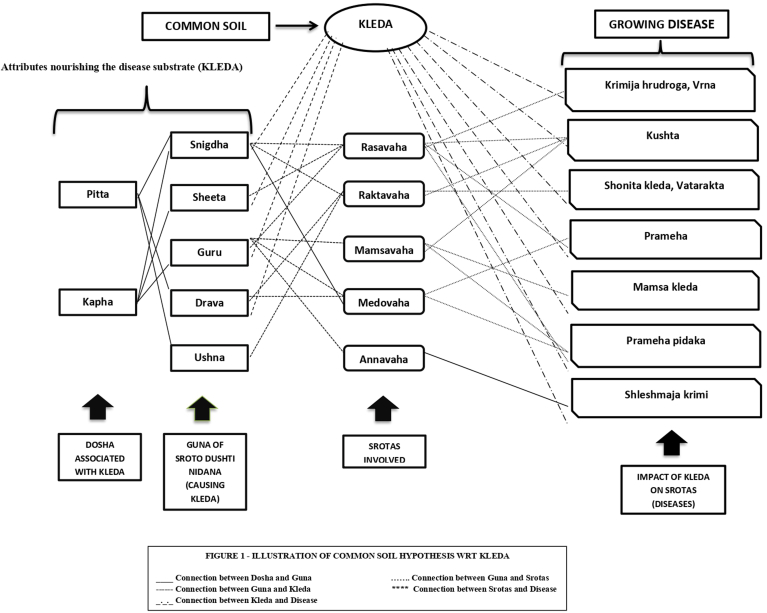
Illustration of common soil hypothesis WRT Kleda.

After *Kleda* sourced from food is expelled without adequately nourishing the essential reserve of bodily tissues, the body resorts to digesting its own tissues for sustenance. This eventually results in a state of depletion known as *Shosha*. This process is akin to what occurs in Diabetes, where weight loss may be triggered by osmotic diuresis due to elevated blood glucose levels.

The Ayurvedic concept of *Kleda* parallels modern understanding concerning fluid equilibrium and elimination, resembling physiological fluids such as blood plasma, lymphatic fluid, and interstitial fluid. These bodily fluids, akin to *Kleda*, sustain tissue hydration, lubrication, and nutrient transport. Furthermore, *Kleda's* role in tissue differentiation mirrors modern concepts of cellular differentiation, repair mechanisms, growth factors, and signalling pathways crucial for maintaining tissue integrity.

Looking at all the diseases involving *Kleda* in their pathogenesis, it becomes evident that they

have a predominance of *Apa Mahabhuta* (termed as ‘*Ap-dhatu* in context of tissues), which may be associated either with *Ushna* (Hot) or *Sheeta* (Cold) *Guna*, as per the dominance of *Pitta* or *Kapha dosha,* respectively.

## 1 Common soil hypothesis

Common soil hypothesis explains the common prevailing substrate, which is invariably involved in the manifestation of several Metabolic disorders. Insulin resistance, vascular inflammation, and endothelial dysfunction all are the projections of “Common soil” [5]. Recent researches demonstrates that inflammation plays principal role in all stages of metabolic disorders, starting from the initial lesion to the end-stage thrombotic complications. Recognising the commonality between the diseases, Stern [6] proposes ‘the common soil hypothesis’ which states that vascular conditions like atherosclerosis which are believed to be complication of diabetes, might have preceded the onset of diabetes and suggests that both conditions may share genetic and environmental antecedents, that is, a ‘common soil’. It has been postulated that chronic low-grade inflammation associated Obesity, insulin resistance, and metabolic abnormalities also acts as ‘common soil’ for Diabetes Mellitus (DM) and Cardio Vascular Disease (CVD) [7]. Glucose intolerance, hyperinsulinemia, dyslipidemia (high triglyceride and low high-density lipoprotein cholesterol levels), and hypertension are the candidates of Insulin Resistance Syndrome. Similarities among these conditions are seen at the level of molecular drivers, pathways, and gene subnetworks. Other inflammation-related mechanisms, such as endothelial dysfunction, atherosclerosis, and increased plaque vulnerability also play key roles. The common soil hypothesis states that Metabolic Syndrome may be considered as surrogate marker and red flag sign for cancer risk among individuals susceptible with unhealthy diet [8].

The concept of “*Kleda*” in Ayurveda shares some parallels with the “Common Soil Hypothesis”, particularly in understanding disease manifestation and the interplay between physiological factors. The Common Soil Hypothesis proposes that various chronic diseases, despite their apparent differences, might have a common origin or underlying factor. Both these concepts are similar in disease manifestation, and play a vital role in role in manifestation of metabolic disorders.

## 2 Clinical application of Kleda concept

**Fig. 2:**
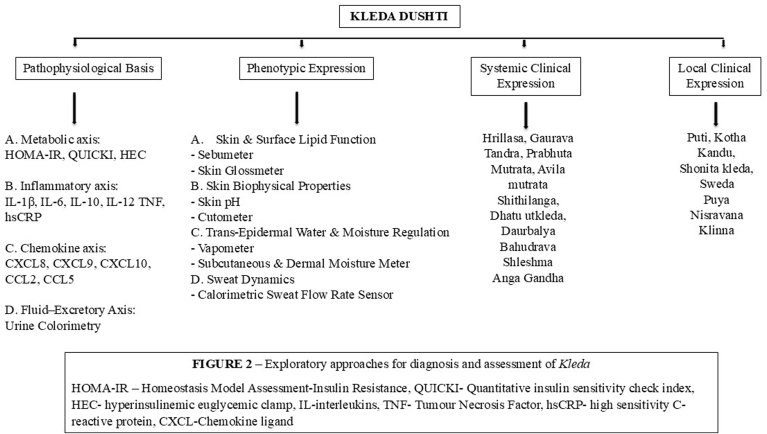
Exploratory approaches for diagnosis and assessment of Kleda.

*Kleda* plays a significant role in the management of *Santarpanotta Vikaras* (diseases with ‘excess tissues’) which universally has dominance of *Guru (∼Heavy), Shita (Cold), Snigdha (Oily), Shlakshna* attributes in causative factors (Charaka Samhita, Sootra 22/78). Nutrition is a key modifiable factor for metabolic syndrome. A study in healthy men suggested that the initial event caused by overnutrition (approx. 6000 kcal/day) causes rapid systemic and adipose tissue insulin resistance, promoting carbonylation and inactivation, mediated by early-onset oxidative stress. [9]. *Kleda,* representing the body's moisture and fluid balance, plays a crucial role in metabolic processes and homeostasis, making its assessment essential for understanding physiological equilibrium. Potential diagnostic approaches may include urine analysis, biochemical markers of hydration and metabolism, and oxidative stress assessments to understand *Kleda's* imbalance, as postulated in Fig. 2. While this hypothesis is proposed, thorough and rigorously designed scientific studies are required to validate these approaches and establish their effectiveness in clinical practice

Food articles with Bitter, Pungent and Astringent taste are said to do *Kleda Upashoshana* (∼Reduce excess dampness) and they are opposite in qualities to the aetiologies causing Metabolic diseases like *Santarpanottha vikara*. Acharya Charaka states activities like strenuous physical exercise and maintaining positive psychological states help reduce *Kleda*. The entire treatment protocol of *Sthoulya* (Obesity) talks in similar words, highlighting on implementation of *Vyayama* (Physical activity), *Manaso Chinta* (indulging in vigil mental actions), *Ruksha chikitsa* (∼Treatment imparting dryness), by using *Tikta* (Bitter), *Katu* (Pungent), *Kashaya* (Astringent) *Rasa Dravyas.* These drugs in general reduces *Kleda* thereby unclogging the *Srotas*. (Charaka Samhita, Sootra 21/21).

**Fig. 3:**
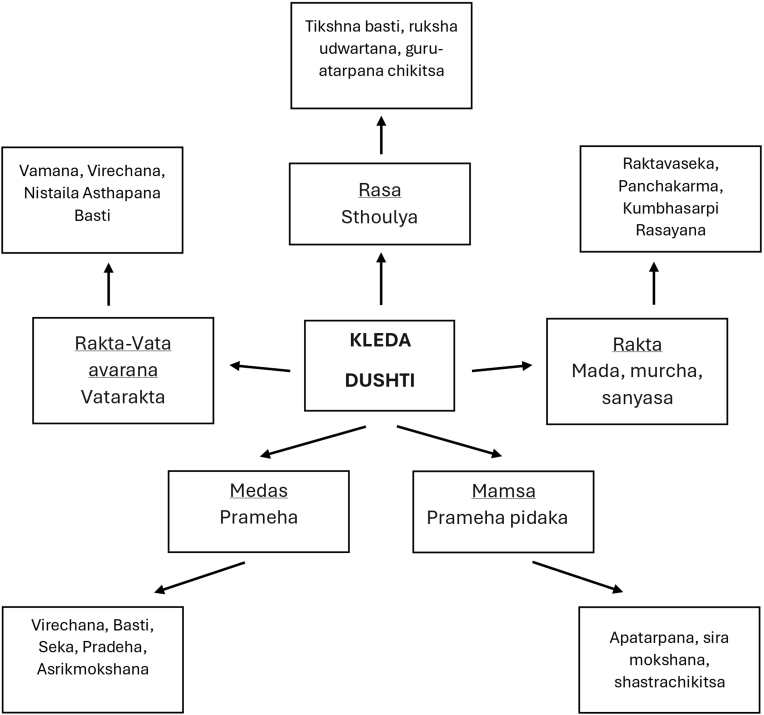
Site specific Kleda and chikitsa.

*Kleda* is an entity in the body which exists in two forms. One when associated with *Shita* (∼Cold) *Guna* of *Kapha Dosha* and other, when associated with *Ushna* (∼hot*) Guna* of *Pitta Dosha.* This association of Cold/Hot attributes decides the pathway, site, severity and treatment strategies for the disease*.* This *Sthana Vishesha* (Site specific) *Kleda* has got varied number of management methods starting from external therapies *(Bahirparimarjana Chikitsa),* Enemas *(Basti Chikitsa), Sanjna Sthapana Chikitsa* (∼Consciousness restorative therapy) and surgical interventions *(Shastrakarma*) in different diseases like *Vatarakta, Mada-Murcha, Prameha, Prameha Pidika* etc, as shown in Fig. 3.

## 3 Conclusion

*Kleda* mentioned in various contexts delineate different understandings. Commonest among all of them is the presence of *Soumya Bhava* or the predominance of *Apa Mahabhoota*. Hence, the term *Kleda* can be inferred as moisture/dampness. Physiologically, *Kleda* has multiple roles in conducting distinct functions of the human body. Pathologically, *Kleda* induces excess *Soumya Bhava* to tissues owing to the abnormal increase in *Apa Mahabhuta*. *Kleda* in the body can manifest in two different states. One is associated with *Sheeta Guna* of *Kapha Dosha* and other being associated with *Ushna Guna* of *Pitta Dosha*. This common substrate of *Kleda* gets nourished by *Drava*, *Snigdha*, *Guru Nidana*s, which are also the reasons for *Santarpanotha Vyadhi*. And this state when persists for a longer time may lead to the sub-acute chronic inflammation owing to the state of oxidative stress. This state can trigger various pathologies further giving rise to the outspread arena of metabolic syndrome. By understanding the wide concept of *Kleda*, we may conclude that it may potentially serve as a surrogate marker for Metabolic Syndrome and its components. Hence understanding and acknowledging the concept of *Kleda* would help in obtaining a holistic approach in the promotion of health, prevention, and management of various forms of metabolic disorder which sprout from the unified soil of *Kleda*.

## Author contributions

NKR: Conceptualization, framework development, data interpretation.

MHS: Literature review, data organization, manuscript drafting, critical revision of the manuscript, and contribution to conceptual refinement.

## Declaration of generative AI in scientific writing

The authors used ChatGPT (OpenAI) solely for language editing, grammar correction, and improving readability of the manuscript. The authors carefully reviewed and verified the content after AI-assisted editing.

## Funding sources

The authors declare that no specific funding was received for this work.

## Declaration of competing interest

The authors declare that there are no conflicts of interest related to this study.
